# Low Serum Superoxide Dismutase Is Associated With a High Risk of Cognitive Impairment After Mild Acute Ischemic Stroke

**DOI:** 10.3389/fnagi.2022.834114

**Published:** 2022-02-28

**Authors:** Ming-Si Zhang, Jian-Hai Liang, Meng-Jia Yang, Yue-Ran Ren, Dai-Hong Cheng, Qi-Heng Wu, Yan He, Jia Yin

**Affiliations:** ^1^Department of Neurology, Nanfang Hospital, Southern Medical University, Guangzhou, China; ^2^Microbiome Medicine Center, Department of Laboratory Medicine, Zhujiang Hospital, Southern Medicine University, Guangzhou, China

**Keywords:** ischemic stroke, cognitive impairment (CI), superoxide dismutase, MMSE, MoCA

## Abstract

**Background:**

Post-stroke cognitive impairment (PSCI) is a common complication after stroke, but effective therapy is limited. Identifying potential risk factors for effective intervention is warranted. We investigated whether serum superoxide dismutase (SOD) levels were related to cognitive impairment after mild acute ischemic stroke (AIS) by using a prospective cohort design.

**Methods:**

A total of 187 patients diagnosed with mild AIS (National Institutes of Health Stroke Scale ≤ 8) were recruited. Serum SOD, erythrocyte sedimentation rate (ESR), C-reactive protein (CRP), and interleukin-6 (IL-6) levels were measured, and cognitive assessments (Mini-Mental State Examination, MMSE; Montreal Cognitive Assessment, MoCA) were performed in the early phase (within 2 weeks). These indexes and assessments were repeated at 3 months after onset. MoCA < 22 was defined as early cognitive impairment (CI-E) within 2 weeks and late cognitive impairment (CI-L) at 3 months after stroke.

**Results:**

In a survey, 105 of 187 (56.1%) patients were identified as CI-E after mild AIS. Lower serum SOD associated with higher inflammatory biomarkers (ESR, CRP, and IL-6) and worse cognitive scores was observed in CI-E patients. In a survey, 39 of 103 (37.9%) stroke patients who completed the 3-month follow-up were identified as CI-L. Serum SOD was consistently lower in CI-L patients at baseline and 3 months and positively associated with cognitive scores. In adjusted analyses, low serum SOD at baseline was independently associated with high risks of CI-E and CI-L, with odds ratios (ORs) of 0.64 and 0.33 per standard deviation increase in serum SOD, respectively. Multiple-adjusted spline regression models showed linear associations between serum SOD and CI-E (*P* = 0.044 for linearity) and CI-L (*P* = 0.006 for linearity). Moreover, 35.2% (19/54) of CI-E patients cognitively recovered during the 3-month follow-up. In multivariable analysis, SOD was identified as a protective factor for cognitive recovery after stroke (OR 1.04, 95% CI: 1.01–1.08, *P* = 0.024).

**Conclusion:**

We demonstrate that low serum SOD is associated with a high risk of cognitive impairment after mild AIS, indicating SOD may be a potential modifiable factor for PSCI.

## Introduction

Post-stroke cognitive impairment (PSCI) is a common complication of stroke as well as a major cause of long-term disability and reduced quality of life. PSCI occurs after 2–6 months in 43.6% of patients with mild acute ischemic stroke (AIS) (Weaver et al., 2021). Unfortunately, no effective medical interventions for PSCI have been recommended to date due to the complex pathogenesis (Quinn et al., 2021). PSCI is currently considered a neurodegenerative disease with progression of both vascular and degenerative lesions. Apart from the pathological features of cerebral parenchyma caused by vascular etiology, potential pathophysiological mechanisms summarized in the consensus report included blood-brain barrier (BBB) breakdown, amyloid cascade, tau hyperphosphorylation, oxidative stress, and inflammation (Bordet et al., 2017). In previous studies, antioxidant stress was proven to reduce post stroke injury and alleviate the progression of neurodegeneration in animal experiments (Margaill et al., 2005; Kim et al., 2020). These results indicated that reduced antioxidant stress ability may be a pathogenic mechanism of PSCI, despite as a multi-etiologic disease.

Superoxide dismutase (SOD) is a group of oxidoreductases that catalyze the dismutation of superoxide radicals into H_2_O_2_ and O_2_, which play a key role in antioxidant stress and anti-inflammation (Fukai and Ushio-Fukai, 2011). SOD deficiency contributes to several neurological diseases, including amyotrophic lateral sclerosis (ALS), Alzheimer’s disease (AD), Parkinson’s disease (PD), and stroke (Flynn and Melov, 2013). An observational study revealed that a dramatic decline in SOD activity after AIS was associated with more severe neurological function and gradually decreased with infarct size (Spranger et al., 1997). Similarly, animal experiments have shown that brain infarction significantly increased after transient middle cerebral artery occlusion (MCAO) in mice with SOD gene mutations or defects (Kim et al., 2002; Xu et al., 2021). Moreover, reduced SOD in plasma or brain related to cognitive impairment was found in patients with schizophrenia, cerebral small vessel disease, and AD (Zhang et al., 2014; Zhu et al., 2019; Fracassi et al., 2021). SOD deficiency has been shown to drive amyloid β protein oligomerization and promote plaque formation and hyperphosphorylation of tau in transgenic mouse models of AD (Li et al., 2004; Melov et al., 2007; Murakami et al., 2011), which are also generally recognized as important mechanisms for PSCI. However, whether the serum level of SOD is a protective factor for PSCI lacks evidence from prospective cohort study.

Thus, we hypothesized that low SOD may be associated with a high risk of cognitive impairment in patients after AIS. We recruited patients with mild AIS for cognitive assessment in the early phase and at 3 months and quantified serum SOD activity levels to analyze the relationship between SOD and cognitive function after stroke. Furthermore, systemic inflammatory biomarkers (erythrocyte sedimentation rate, ESR; C-reactive protein, CRP; interleukin-6, IL-6) were measured for further insight into the association between SOD, cognitive function, and inflammatory biomarkers in patients with AIS.

## Materials and Methods

### Study Design and Participants

**FIGURE 1:**
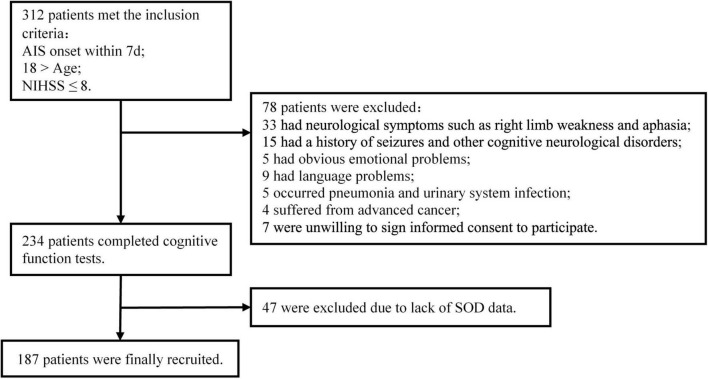
Patient flowchart.

A cohort of patients with mild AIS was established. Patients who met the criteria were enrolled and cognitively assessed using the Mini-Mental State Examination (MMSE) and Montreal Cognitive Assessment (MoCA) in the early phase (within 2 weeks) and at 3 months after stroke, and their serum SOD and systemic inflammatory biomarkers (ESR, CRP, and IL-6) were measured. Clinical data were recorded during hospitalization for analysis. Participants diagnosed with AIS were enrolled in the Department of Neurology at Nanfang Hospital from September 2018 to December 2020 if they met the following criteria: (1) aged 18–75 years old, (2) admitted within 7 days of stroke onset, and (3) National Institutes of Health Stroke Scale (NIHSS) score at admission ≤ 8. The exclusion criteria included the following: (1) failure to complete cognitive assessments due to neurological deficits, such as drowsiness (NIHSS 1a. Level of Consciousness ≥ 1), aphasia (NIHSS Best Language ≥ 1), or right limb weakness; (2) history of seizures and obvious prestroke cognitive impairment (AD8 ≥ 2); (3) history of mental disorders or significant emotional problems; (4) language barrier or failure to cooperate in completing the test; (5) obvious infectious diseases such as pneumonia and urinary system infection; (6) advanced cancer according to their medical histories; (7) lack of SOD data; and (8) unwillingness to sign informed consent to participate. A total of 187 AIS patients met the inclusion criteria and were finally recruited. Details of the enrollment process are shown in Figure 1. The Ethical Committee of Southern Medical University approved all aspects of this study, and informed consent for data collection was obtained from all subjects or their legal guardians.

This study was approved by the Ethics Committee of Nanfang Hospital, Southern Medical University (NFEC-2020-169) and registered at http://clinicaltrials.gov (NCT04688138).

### Cognitive Assessment

The AD8 contains eight questions asking the informant to rate change (Yes vs. No) in memory, problem-solving abilities, orientation, and daily activities. Patients were screened with AD8 before enrollment. AD8 ≥ 2 was considered to indicate prestroke cognitive impairment (Dong et al., 2013). MMSE and MoCA were used for cognitive assessment. The MMSE and MoCA scores ranged from 0 to 30, with higher scores indicating better cognitive function. The MoCA scale evaluates the following seven cognitive domains: visuospatial/executive functions, naming, memory, attention, language, abstraction, and orientation. MoCA and MMSE are widely used tools for global cognitive function assessment after stroke. The feasibility and accuracy of MoCA as a screening tool after stroke have also been verified by previous studies (Horstmann et al., 2014; Lees et al., 2014). Moreover, the MoCA scale can detect complex cognitive impairments, such as executive function and visual perception/construction function, and is more sensitive to the detection of PSCI (Dong et al., 2010). For reasonable sensitivity and specificity of identification, MoCA scores less than 22 were defined as cognitive impairment (Lees et al., 2014). To correct for education effects, one point was added to the total MoCA scores of patients who had less than 12 years of education (Nasreddine et al., 2005). Cognitive assessments were conducted by two trained neurologists and conformance tests were carried out before the study initiated to ensure the reliability of the assessments. MoCA < 22 was defined as early cognitive impairment (CI-E) within 2 weeks and late cognitive impairment (CI-L) at 3 months after stroke. In addition, to analyze the relationship between SOD and cognitive function recovery, we defined patients with CI in the early phase whose MoCA scores recovered to or above the threshold value (22 points) at 3 months as the cognitive recovery group (CR) and those whose MoCA score remained lower than the threshold value as the cognitive non-recovery group (nCR).

### Biochemical Analysis of Blood Samples

The first fasting blood samples were drawn for SOD, inflammatory biomarkers (ESR, CRP, IL-6) at baseline and repeated at 3 months later. Serum SOD activity was determined by pyrogallol autoxidation (Superoxide dismutase Detection Kit, Fuyuan, Fujian, China). The ESR was measured using an automatic ESR analyzer (Electa Lab, XC-40B, Forli, Italy). Immunonephelometry measurement of CRP was performed on a Nephelometer Analyzer BN II (Siemens, Marburg, Germany). IL-6 was detected by enzyme-linked immunosorbent assay (ELISA) using kits (Elecsys IL-6 kit, Roche Diagnostics, Shanghai, China).

### Statistical Analysis

The results are expressed as numbers (percentages, %) for categorical variables and medians (interquartile ranges) for continuous variables. Categorical variables were compared by the χ^2^ test or Fisher exact test, and continuous variables were compared by the Mann-Whitney *U* test. Logistic regression analysis estimated the relationship between SOD and the risk of cognitive impairment after stroke by calculating odds ratios (ORs) and 95% confidence intervals (95% CIs). Multiple adjusted logistic regression models were performed thereafter. Model 1 was unadjusted. Model 2 was adjusted for the clinical features that may affect the cognitive function, including age, sex, education, history of smoking, drinking, hypertension, diabetes, stroke and hyperlipidemia, stroke causes, NIHSS score, Barthel index (BI), body mass index (BMI), and infarct location. Model 3 was adjusted for the factors applied in model 2 as well as factors that may have impact on both cognitive function and serum SOD levels, including WBC, IL-6, ESR, and CRP. ORs of cognitive impairment after stroke were also calculated per standard deviation (SD) increment in serum SOD levels.

Spline regression models were used to provide and explore the shape of the association between serum SOD and cognitive impairment after stroke, fitting a restricted cubic spline function with 4 knots (at the 5th, 35th, 65th, and 95th percentiles). Wald statistics were used to test the non-linear relationship between SOD and cognitive impairment after stroke. Furthermore, we tested the linear trend by entering the median of the quartiles into the model as a continuous variable. A 2-tailed *P*-value < 0.05 was considered statistically significant. R software (package “ggplot” and “rms”), version 3.6.3 (R Core Team, 2019) and SPSS 26 (IBM SPSS, Chicago, IL, United States) were used for all data analyses.

## Results

### Baseline Characteristics

A total of 187 mild AIS patients (median age 56 years; 79.1% male; median NIHSS score 2) were finally recruited. Among them, 105 (56.1%) patients were categorized as early cognitive impairment after AIS (CI-E). Compared with patients without cognitive impairment (nCI-E), the CI-E group was more likely to be older, female, less educated, diabetic, and less likely to be alcohol drinkers. No significant difference was found in BMI, NIHSS scores, BI, stroke cause, or other medical history. Laboratory findings showed that systemic inflammation biomarkers (ESR, CRP, and IL-6) were significantly higher in the CI-E group (Table 1), but there was no distinct difference in the count of total WBC and their subclasses. Furthermore, neuroimaging showed that lacunes and enlarged perivascular spaces (EPVS) were more common in patients with CI-E (Supplementary Table 1).

**TABLE 1:** Baseline characteristics of the recruited patients.

Characteristics*	CI-E	nCI-E	*P*
No. of subjects	105 (56.1)	82 (43.9)	
**Demographics**			
Age, y	59 (13)	54 (16)	0.001
Female	27 (25.7)	12 (14.6)	0.064
Education, y	7 (3)	8 (2)	0.000
Cigarette smoking	55 (52.4)	47 (57.3)	0.501
Alcohol drinking	23 (21.9)	29 (35.4)	0.041
**Medical histories**			
History of hypertension	69 (65.7)	58 (70.7)	0.466
History of hyperlipidemia	38 (36.2)	29 (35.4)	0.907
History of diabetes	43 (41.0)	23 (28.0)	0.067
History of stroke	55 (52.4)	47 (46.1)	0.501
**Clinical features**			
BMI, kg/m^2^	24.2 (4.2)	24.3 (4.2)	0.722
Baseline NIHSS score	2 (4)	2 (2)	0.859
Baseline BI	80 (35)	85 (35)	0.517
Infarcted location (Cortical infarct)	33 (31.4)	29 (35.4)	0.570
Stroke causes (LAA)	65 (61.9)	43 (52.4)	0.193
**Baseline laboratory findings**			
WBC, ×10^9^/L	7.6 (2.8)	7.8 (3.1)	0.799
NEU, ×10^9^/L	5.0 (2.2)	4.6 (2.8)	0.459
LYM, ×10^9^/L	2.0 (0.9)	1.9 (1.2)	0.909
ESR, mm/h	10 (12)	6 (9)	0.000
IL-6, pg/mL	3.54 (3.91)	2.65 (2.63)	0.001
CRP, mg/L	1.37 (2.48)	0.90 (2.50)	0.044
**Cognitive scores within 2 weeks**			
MMSE	24 (5)	28 (2)	<0.001
MoCA	16 (7)	24 (3)	<0.001

*Continuous variables are expressed as median (IQR). Categorical variables are expressed as frequencies (percentages).

*CI-E, early cognitive impairment after stroke; nCI-E, non-early cognitive impairment after stroke; LAA, large-artery atherosclerosis; WBC, white blood cells count; NEU, neutrophil count; LYM, lymphocyte count; ESR, erythrocyte sedimentation rate; CRP, C-reactive protein; IL-6, interleukin-6.*

As summarized in Table 2, the MMSE and MoCA scores in the early phase of the CI-E group were significantly lower than those without cognitive impairment (CI-E vs. nCI-E: MMSE 24 vs. 28, MoCA 16 vs. 24; comparisons here are reported as medians, all *p* < 0.001).

**TABLE 2:** Associations between serum superoxide dismutase (SOD) levels at baseline and cognitive impairment after stroke.

	Model 1	Model 2	Model 3
	OR (95% CI)	OR (95% CI)	OR (95% CI)
**CI-E: MoCA within 2 weeks**			
Per 1 SD increased in SOD	0.62 (0.46–0.87)	0.62 (0.42–0.91)	0.64 (0.42–0.99)
*P*-value	0.005	0.013	0.045
**CI-L: MoCA at 3 months**			
Per 1 SD increased in SOD	0.52 (0.33–0.82)	0.34 (0.17–0.69)	0.33 (0.16–0.72)
*P*-value	0.005	0.003	0.005

*Model 1: unadjusted.*

*Model 2: adjusted for age; sex; education; history of smoking; drinking; hypertension; diabetes; stroke and hyperlipidemia; stroke causes; NIHSS score; Barthel index; BMI; infarcted location.*

*Model 3: adjusted for the factors above, plus WBC; ESR; IL-6 and CRP.*

*1 SD = 23.0 U/ml.*

### Lower Serum Superoxide Dismutase Is Correlated With Worse Cognitive Scores and Higher Systemic Inflammation Biomarkers

**FIGURE 2:**
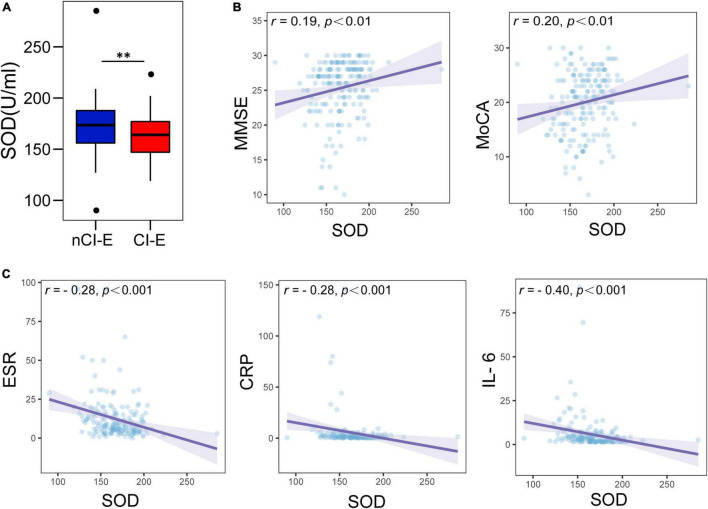
Comparison of baseline serum superoxide dismutase (SOD) levels and association between SOD and cognitive scores, systemic inflammation biomarkers. **(A)** Comparison of SOD levels in the CI-E group and NCI-E group. **(B)** Associations between serum SOD and cognitive scores (MMSE and MoCA). **(C)** Associations between serum SOD and systemic inflammation biomarkers (ESR, CRP, and IL-6). CI-E, early cognitive impairment after stroke; nCI-E, non-early cognitive impairment after stroke. ^**^P < 0.01.

In comparison of the serum SOD between CI-E group and nCI-E group, we found that the median serum SOD levels at baseline in CI-E group was 164 U/ml, which was significantly lower than that in nCI-E group (174 U/ml) (Figure 2A). In the spearman correlation analysis, lower SOD levels were associated with worse cognitive scores (MMSE, *r* = 0.19, *P* < 0.01; MoCA, *r* = 0.20, *P* < 0.01, Figure 2B). Moreover, SOD levels were inversely associated with systemic inflammatory biomarkers (ESR, *r* = −0.28, *P* < 0.001; CRP, *r* = −0.28, *P* < 0.001; IL-6, *r* = 0.40, *P* < 0.001; Figure 2C), which have been reported as risk factors for PSCI (Kliper et al., 2013; Zhang and Bi, 2020). These results suggest that low SOD levels accompanied with high systemic inflammation may be important symbols for cognitive impairment in the early phase after mild AIS.

### Serum Superoxide Dismutase Is Consistently Lower in CI-L Patients

At 3 months after onset, 103 of stroke patients completed follow-up and cognitive assessment. In a survey, 39 (37.9%) of them were identified as CI-L, including 35 CI-E patients and 4 nCI-E patients. CI-L patients were more likely to be female, older and less educated. The cognitive scores (MMSE, MoCA) at baseline and 3 months were significantly lower in CI-L patients. And there were no difference in medication between two group (Supplementary Tables 2, 3).

**FIGURE 3:**
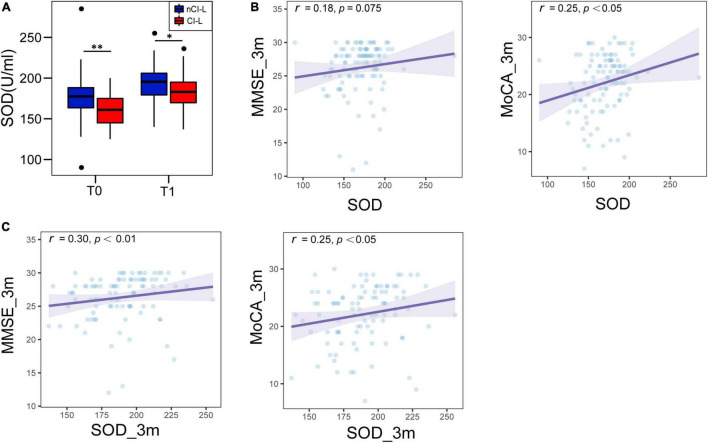
Comparison of serum SOD levels between CI-L and nCI-L and the association between SOD and cognitive scores at follow-up. **(A)** Comparison of SOD levels (both baseline and 3-month follow-up) in the CI-L group and nCI-L group. **(B)** Associations between serum SOD (baseline) and cognitive scores (MMSE and MoCA at follow-up). **(C)** Associations between serum SOD (follow-up) and cognitive scores (MMSE and MoCA at follow-up). CI-L, late cognitive impairment after stroke; nCI-L, non-late cognitive impairment after stroke. *P < 0.05, ^**^P < 0.01.

Moreover, systemic inflammatory markers (ESR and IL-6) were significantly higher in patients with CI-L at baseline and follow-up periods than in those without cognitive impairment (nCI-L. Supplementary Tables 2, 3). And serum SOD levels were consistently lower in the CI-L patients (medians in the CI-L group vs. nCI-L group: 177 U/ml vs. 160.5 U/ml at baseline, *P* < 0.01; 195.5 U/ml vs. 183 U/ml at follow-up, *P* < 0.05), though they tended to increase at 3 months of follow-up (Figure 3A). These results suggested persistent low levels of SOD and chronic low-grade inflammation in CI-L patients. In spearman correlation analysis, we found that cognitive scores at 3 months were positively correlated with serum SOD levels at baseline (MMSE, *r* = 0.18, *P* = 0.075; MoCA, *r* = 0.25, *P* < 0.05, Figure 3B) and at 3 months (MMSE, *r* = 0.30, *P* < 0.01; MoCA, *r* = 0.25, *P* < 0.05, Figure 3C).

### Low Serum Superoxide Dismutase Is a Risk Factor of Cognitive Impairment After Stroke

Next, logistic regression analysis was used to explore the relationship between baseline serum SOD levels and cognitive impairment after stroke. As summarized in Table 2, per SD (23.0 U/mL) increased in baseline serum SOD levels had ORs of 0.63 (95% CI: 0.46–0.87, *P* = 0.005; Model 1) for CI-E and 0.52 (95% CI: 0.33–0.82, *P* = 0.005; Model 1) for CI-L.

**FIGURE 4:**
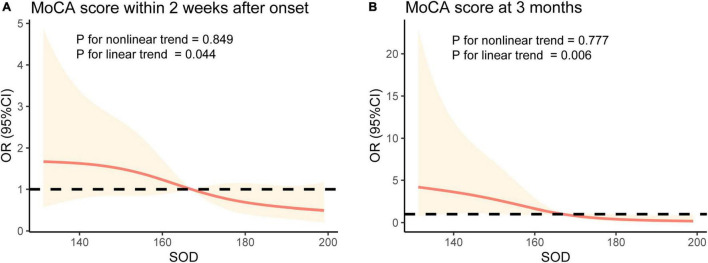
Associations between serum SOD levels and cognitive impairment after stroke using restricted cubic spline regression models. The OR for cognitive impairment in early phase **(A)** and at 3 month **(B)** decreased with serum SOD. ORs were adjusted for the same variables as Model 3 in Table 2.

In analyses adjusted for age, sex, education, history of smoking, drinking, hypertension, diabetes, stroke and hyperlipidemia, stroke causes, NIHSS score, BI, BMI, and infarct location (Model 2), the patients with high serum SOD had a low risk of CI-E (OR 0.62, 95% CI: 0.42–0.91, *P* = 0.013) and CI-L (OR 0.34, 95% CI: 0.17–0.69 per SD, *P* = 0.003). After further adjustment for WBC, ESR, IL-6, and CRP (Model 3), the per-SD increases in SOD had ORs of 0.64 (95% CI: 0.42–0.99, *P* = 0.045) and 0.33 (95% CI: 0.16–0.72, *P* = 0.005) for CI-E and CI-L, respectively (Table 2). The associations persisted when further adjusted for lacunes, Fazekas scores, and EPVS (CI-E: OR 0.49, 95% CI: 0.27–0.90, *P* = 0.020; CI-L: OR 0.26, 95% CI: 0.08–0.83, *P* = 0.023). Multiple-adjusted spline regression models showed the dose-response relationships between baseline SOD levels and cognitive impairment in the early phase and at 3 months after onset. Tests for linear trends further confirmed a linear relationship between baseline SOD levels and cognitive impairment in the early phase (*P* = 0.044 for linearity; Figure 4A) and at 3 months (*P* = 0.006 for linearity; Figure 4B).

### Serum Superoxide Dismutase Is Associated With Cognitive Rehabilitation After Stroke

**FIGURE 5:**
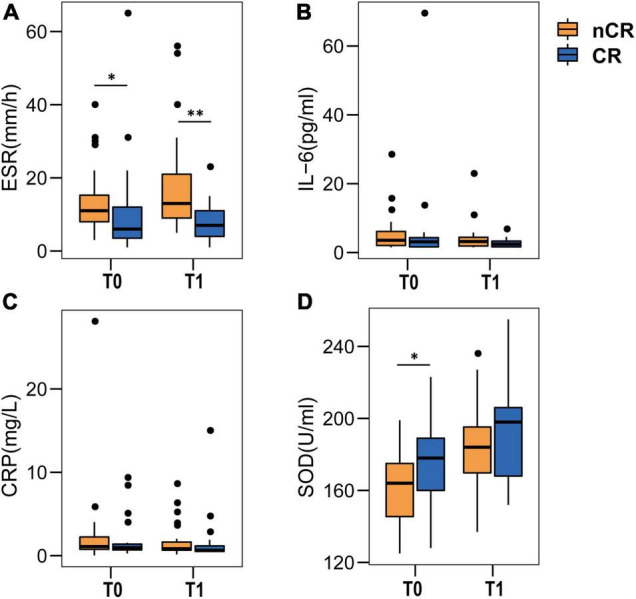
Comparison of systemic inflammatory biomarkers and serum SOD levels between CR and nCR. **(A–C)** Comparison of systemic inflammation biomarkers. Compared to the CR group, ESR, CRP, and IL-6 **(A–C)** increased in the nCR group at baseline (T0), especially ESR levels, which were consistently (T0 and T1) higher in the nCR group **(A)**. **(D)** Comparison of serum SOD levels. SOD was continuously lower in the nCR group, especially at baseline. T0 means baseline, T1 means 3-month follow-up. ESR, IL-6, CRP, and SOD at baseline and 3 months were compared between two groups by Mann-Whitney *U* test. *P < 0.05, ^**^P < 0.01.

In this part, we attempted to explore the relationship between SOD and cognitive rehabilitation by observing the transformation of cognitive status in patients with early cognitive impairment after stroke (CI-E group, a total 54 of CI-E group completed a 3-month cognitive assessment). We found that 35.2% (19/54) of the patients with cognitive impairment cognitively recovered during follow-up (CR group), while 64.8% (35/54) of CI-E patients maintained cognitive impairment (nCR group). The ESR was consistently higher in the nCR group than in the CR group (medians in the nCR group vs. CR group: 11 vs. 6 mm/h at baseline, *P* < 0.05; 13 vs. 7 mm/h at 3 months, *P* < 0.01; Figure 5A). IL-6 and CRP were also slightly higher than CR group, but not significantly (Figures 5B,C). Moreover, SOD levels were continuously lower in the nCR group than in the CR group (median SOD levels in the nCR group vs. CR group: 164 U/mL vs. 178 U/mL at baseline, *P* < 0.05; 184 U/mL vs. 198 U/mL at 3 months, *P* > 0.05; Figure 5D). Furthermore, SOD was identified as an independent protective factor for cognitive recovery in univariate (OR 1.03, 95% CI: 1.00–1.06, *P* = 0.035) and multivariable regression analyses (OR 1.04, 95% CI: 1.01–1.08, *P* = 0.024) after adjusting for confounders (age, sex, education, BMI, baseline NIHSS score, BI, ESR, CRP, and IL-6) (Table 3).

**TABLE 3:** Associations between serum SOD levels at baseline and cognitive rehabilitation after stroke among patients with CI.

Variables adjusted	OR (95% CI)	*P*-value
Unadjusted	1.03 (1.00–1.06)	0.035
Demographics	1.04 (1.01–1.08)	0.023
Demographics + Neurological scores	1.04 (1.01–1.08)	0.021
Demographics + Neurological scores + Inflammatory biomarkers	1.04 (1.01–1.08)	0.024

*Demographics included age, sex, education, BMI. Neurological scores included baseline NIHSS score, BI. Inflammatory biomarkers included ESR, IL-6, and CRP. In this part, limited by the number of patients, we only considered the above factors that may have a significant impact on cognitive function when adjusting confounding factors.*

## Discussion

In our data, serum SOD levels were significantly lower in patients with cognitive impairment in the early phase and at 3 months after mild AIS than in those without cognitive impairment, accompanied by increased systemic inflammation biomarkers (ESR, CRP, and IL-6). Cognitive scores in the early phase and at 3 months after onset were positively correlated with serum SOD levels. Multivariable analyses showed that low serum SOD levels were associated with a higher risk of cognitive impairment after stroke. High levels of SOD might be one of the protective factors for cognitive rehabilitation after stroke. These findings suggested that low SOD may be a new risk factor for cognitive impairment and cognitive rehabilitation after stroke.

To date, three isoforms of superoxide dismutases have been biochemically and molecularly characterized in mammals, including SOD1 (Cu/ZnSOD) in cytoplasm, SOD2 (MnSOD) in mitochondrial matrix and SOD3 (ecSOD) in extracellular matrix, cell surface, and extracellular fluid. SOD1 and SOD2 are expressed in practically all cells, while SOD3 was identified in selected tissues, particularly in blood vessels, lung, kidney, and heart, as well as extracellular fluids including the blood (Zelko et al., 2002; Wang et al., 2018). In the current study, pyrogallol autoxidation method was used to measure the total SOD activity in serum indirectly, which could not differentiate between the different SOD isoforms (Marklund and Marklund, 1974). However, according to the specific distribution, the SOD activity was speculated to be mainly derived from SOD3. In our data, the correlation coefficients between SOD and cognitive score (MMSE, MoCA) were not high, and the differences in inflammatory indexes (ESR, CRP, IL-6) between patients with and without cognitive impairment were small. It may be explained by the complicated etiology or risk factors of PSCI, while SOD deficiency and inflammation may only contribute part of the pathogenesis to PSCI. If the relationship between SOD and PSCI can be clarified, SOD supplement may also be one of potential target for intervention. Moreover, the small difference of inflammatory indexes suggested that patients with cognitive impairment were experiencing a low-grade inflammation, which was found to be associated with cognitive decline and increased risk of mortality during a long-term follow-up (Karlsson et al., 2010).

Cognitive function sharply declined following a stroke event (Zheng et al., 2019). Apart from stroke injury, cognitive impairment after acute stroke was closely associated with cognitive reserve and brain reserve (Umarova, 2017). The reduced SOD in patients with early cognitive impairment may be related to pathophysiological changes pre stroke. Previous studies found that SOD deficiency accelerates amyloid β oligomerization and memory loss in AD model mice (Murakami et al., 2011). Overexpression of mitochondrial MnSOD could reduce the synthesis of Aβ and prevent the neural apoptosis induced by Aβ (Keller et al., 1998). Moreover, SOD deficiency also promoted the phosphorylation of tau protein in a mouse model of AD (Melov et al., 2007). And postmortem autopsy found that SOD levels were lower in the brain neurons of AD patients (Fracassi et al., 2021). These clues suggested that oxidative stress promotes the progression of AD-related pathological processes, which might be one of the key pathways by which SOD deficiency affects cognitive status prestroke. In addition, SOD deficiency also contributed to accelerate vascular injury and BBB breakdown. In mouse models of ALS-causing SOD1 mutants, deficiency of SOD1 reduced the levels of the tight junction proteins ZO-1, occludin and claudin-5 between endothelial cells and destroyed the blood-spinal barrier, which resulted in microbleedings and hypoperfusion of microcirculation (Zhong et al., 2008). BBB leakage was found to be dose-dependent on copper/zinc (CuZn)-SOD in a mouse model of traumatic brain injury (Mikawa et al., 1996). SOD supplementation could attenuate increased BBB permeability after ischemia in piglets (Armstead et al., 1992). These results suggest that SOD deficiency accelerates vascular injury and the BBB breakdown might be one of the mechanisms leading to neuronal injury and cognitive impairment. Furthermore, reduced SOD levels were correlated with cerebral small vessel diseases. Patients with fewer WMHs had higher plasma SOD levels and better cognitive performance (Zhu et al., 2019). These clues suggested that the reduction in SOD in early stroke may be related to the change in pre stroke brain reserve, caused cognitive impairment in early stroke.

The continuous reduction in SOD may be involved in cognitive impairment after stroke through the following mechanisms. First, SOD was correlated with size of infarction. An observational study showed consistent results that serum SOD levels inversely correlated with the size of infarction and the severity of neurological deficits in AIS patients (Spranger et al., 1997). The damage to brain tissue caused by MCAO was significantly alleviated by supplementation with exogenous SOD or its mimics (Huang et al., 2012; Yun et al., 2013; Dohare et al., 2014). These results suggested that the reduced SOD may exacerbate brain injury after stroke, which may contribute to cognitive impairment. Furthermore, the reduced SOD weakens the ability of antioxidant stress and anti-inflammatory, and the stronger inflammatory response after stroke leads to secondary injury in the brain. Consistent with this, low SOD levels in CI-L patients were accompanied by a continuous increase in systemic inflammatory biomarkers (ESR, IL-6, and CRP) in our data. These inflammatory biomarkers have been recognized as risk factors for PSCI (Kliper et al., 2013; Zhang and Bi, 2020).

The cognitive score of patients with stroke increased rapidly during the first 3 months and continued for 1 year (Sagnier et al., 2019; Shin et al., 2020), which could be promoted by education and occupation (Shin et al., 2020), but brain injury such as chronic cortical cerebral microinfarcts could slow recovery (Sagnier et al., 2019). In our data, serum SOD levels were higher in patients with recovery from CI-E and were independently associated with cognitive recovery after adjusting for confounders. However, the ORs were relatively small, which could be explained by the variation in SOD levels, and the OR value represented the effect of every increased unit of serum SOD on the outcome. The relationship between SOD and cognitive recovery may be explained by higher SOD being associated with better brain reserve.

Though SOD deficiency is widely recognized correlated with cognitive impairment, there are some conflicting results. In a longitudinal cohort of elderly adults, data showed that the highest quartile of SOD activity had a 1.32 times higher risk of cognitive decline than the lowest quartile (Sun et al., 2019). SOD activity inversely correlated with cognitive scores assessed by the Repeatable Battery for the Assessment of Neuropsychological Status was also found in schizophrenia patients (Wu et al., 2014). Furthermore, in patients with Down syndrome, symptoms of Alzheimer’s-like dementia demonstrated in some studies might be attributed to overexpression of SOD, which stems from triplication of chromosome 21 (Perluigi and Butterfield, 2011). There are several explanations for these phenomena. The increased activity of SOD leads to the overproduction of H_2_O_2_, which at high, toxic levels may be responsible for neuronal damage/loss (Perluigi and Butterfield, 2011). Excess H_2_O_2_ further altered the redox environment and compromised the function of the N-methyl-D-aspartate receptor (Lee et al., 2014). However, the etiology of these cognitive impairments differs from that of PSCI, which may cause different SOD manifestations.

There were several limitations in this study. First, this was a short-term follow-up study with a small sample size in a single center, which may suffer from limited statistical power and may not be generalizable. Due to the wide medical coverage of the center, part of patients refused to complete the cognitive assessment follow-up, resulting in longer-term follow-up data was not obtain. Second, limited by the current cognitive assessment methods, patients with moderate to severe stroke and aphasia could not fully cooperate with scale evaluation due to neurological deficits, which may lead to the underestimate of their cognitive function. Thus, we only conducted cognitive assessment on patients with mild stroke. The generalizability of the results to patients with moderate to severe stroke remains to be confirmed. Furthermore, we excluded only patients with significant mood disorders, and no further mental assessment was performed to observe the effect of emotional state. Finally, the underlying mechanism was still unclear and needed to be explored by rigorous experiments in the future.

### Summary

In summary, this study revealed that lower SOD levels in patients with cognitive impairment after stroke were accompanied by increased systemic inflammation biomarkers. Low serum SOD was associated with a high risk of cognitive impairment after mild AIS, indicating SOD may be a potential modifiable factor for PSCI. These clues would help to gain the further insights into pathophysiology of PSCI.

## Data Availability Statement

The original contributions presented in the study are included in the article/Supplementary Material, further inquiries can be directed to the corresponding authors.

## Ethics Statement

The studies involving human participants were reviewed and approved by the Ethics Committee of Nanfang Hospital, Southern Medical University. The patients/participants provided their written informed consent to participate in this study. Written informed consent was obtained from the individual(s) for the publication of any potentially identifiable images or data included in this article.

## Author Contributions

M-SZ, J-HL, YH, and JY designed the study. M-SZ, J-HL, and JY performed and supervised the study. M-SZ, J-HL, and YH performed and analyzed all the data. M-SZ and Q-HW performed the cognitive assessments. M-JY, Y-RR, and D-HC collected blood samples and clinic data. M-SZ wrote the manuscript. M-SZ, YH, and JY conceived the study, supervised the participants, and revised the manuscript. All authors contributed to the article and approved the submitted version.

## Conflict of Interest

The authors declare that the research was conducted in the absence of any commercial or financial relationships that could be construed as a potential conflict of interest.

## Publisher’s Note

All claims expressed in this article are solely those of the authors and do not necessarily represent those of their affiliated organizations, or those of the publisher, the editors and the reviewers. Any product that may be evaluated in this article, or claim that may be made by its manufacturer, is not guaranteed or endorsed by the publisher.
