# Supplementary material for: Low Serum Superoxide Dismutase Is Associated With a High Risk of Cognitive Impairment After Mild Acute Ischemic Stroke
Source: Front Aging Neurosci. 2022 Feb 28;14:834114. doi: 10.3389/fnagi.2022.834114 (PMC8920119; doi:10.3389/fnagi.2022.834114)
Supplement: Supplementary file 1 [file Table_1.doc]

**Low Serum Superoxide Dismutase Is Associated With a High Risk of**

**Cognitive Impairment After Mild Acute Ischemic Stroke**

***Supplementary Materials***

**Supplementary Table 1**. Magnetic resonance imaging (MRI) findings between patients with cognitive impairment and those without cognitive impairment

|  | CI-E | nCI-E | P | CI-L | nCI-L | P |
| --- | --- | --- | --- | --- | --- | --- |
| **MRI finding** | **n=84** | **n=64** |  | **n=31** | **n=52** |  |
| **WMH** |  |  |  |  |  |  |
| PVWMH | 1(1) | 1(1) | 0.169 | 1(1) | 1(1) | 0.015 |
| DWMH | 1(0) | 1(1) | 0.308 | 1(0) | 1(1) | 0.033 |
| Fazekas score | 2(2) | 2(2) | 0.283 | 2(1) | 2(2) | 0.010 |
| **Lacune** | 57 (67.9) | 30 (46.9) | 0.010 | 23 (74.2) | 23 (43.2) | 0.008 |
| **EPVS** | 37 (44.0) | 19 (29.7) | 0.074 | 14 (45.2) | 15 (28.8) | 0.132 |

Continuous variables are expressed as median (IQR). Categorical variables are expressed as frequencies (percentages).

WMH, White Matter Hyperintensities; PVWMH, Periventricular White Matter Hyperintensities; DWMH, Deep White Matter Hyperintensities; EPVS, Enlarged Perivascular Space.

The Fazekas Scale was used to rate the degree of white matter hyperintensity (Fazekas, et al., 1987).

REFERENCE

Fazekas F, Chawluk JB, Alavi A, Hurtig HI, Zimmerman RA. (1987). Mr Signal Abnormalities at 1.5 T in Alzheimer's Dementia and Normal Aging*. AJR Am J Roentgeno*l 149, 351-356. doi: 10.2214/ajr.149.2.351

**Supplementary Table 2.** **Baseline characteristics and laboratory findings of patients completed follow-up**

| **Characteristics** | CI-L | nCI-L | | P |
| --- | --- | --- | --- | --- |
| **No. of subjects**  **Demographics** | 39 (37.9) | 64 (62.1) | |  |
|  |  |  | |
| Age, y | 61 (12) | 54 (15) | | 0.001 |
| Female | 13 (33.3) | 8 (12.5) | | 0.011 |
| Education, y | 5 (2) | 8 (2) | | < 0.001 |
| Cigarette smoking | 18 (46.2) | 41 (64.1) | | 0.075 |
| Alcohol drinking | 7 (17.9) | 20 (31.3) | | 0.137 |
| **Medical histories** |  |  | |  |
| History of Hypertension | 28 (71.8) | 43 (67.2) | | 0.624 |
| History of hyperlipidemia | 14 (35.9) | 21 (32.8) | | 0.748 |
| History of Diabetes | 16 (41.0) | 20 (31.3) | | 0.313 |
| History of stroke | 7 (17.9) | 5 (7.8) | | 0.120 |
| **Clinical features** |  |  | |  |
| BMI, kg/m^2^ | 23.7 (3.9) | 23.9 (3.8) | | 0.399 |
| Baseline NIHSS score | 2 (3) | 2 (2) | | 0.568 |
| Baseline BI | 75 (40) | 85 (39) | | 0.557 |
| Infarcted location  (Cortical infarct) | 15 (38.5) | 20 (31.3) | | 0.454 |
| Stroke causes (LAA) | 22 (56.4) | 38 (59.4) | | 0.767 |
| **Baseline laboratory findings** |  |  | |  |
| WBC, × 10^9^/L | 7.5 (2.4) | 7.8 (2.8) | | 0.708 |
| NEU, × 10^9^/L | 4.9 (1.9) | 4.7 (2.7) | | 0.801 |
| LYM, × 10^9^/L | 1.9 (0.8) | 1.9 (1.2) | | 0.644 |
| ESR, mm/h | 11 (8) | 6 (7) | | 0.001 |
| IL-6, pg/mL | 3.59 (5.20) | 2.56 (2.47) | | 0.018 |
| CRP, mg/L | 1.11 (1.94) | 0.89 (1.91) | | 0.301 |
| **Cognitive scores within 2 weeks** |  |  | |  |
| MMSE | 23 (6) | 28 (3) | | < 0.001 |
| MoCA | 13 (6) | 23 (4.5) | | < 0.001 |

Continuous variables are expressed as median (IQR). Categorical variables are expressed as frequencies (percentages).

CI-L, late cognitive impairment after stroke; nCI-L, non-late cognitive impairment after stroke; LAA, large-artery atherosclerosis; WBC, white blood cells count; NEU, neutrophil count; LYM, lymphocyte count; ESR, erythrocyte sedimentation rate; CRP, C-reactive protein; IL-6, interleukin-6.

**Supplementary Table 3. Laboratory, cognitive scores and medications during follow-up**

|  | CI-L | nCI-L | P |
| --- | --- | --- | --- |
| **Laboratory findings at 3 months** |  |  |  |
| ESR, mm/h | 13 (13) | 9 (10) | 0.002 |
| IL-6, pg/mL | 3.18 (3.02) | 2.11 (1.45) | 0.005 |
| CRP, mg/L | 0.84 (1.37) | 0.64 (1.25) | 0.065 |
| **Cognitive scores at 3 months** |  |  |  |
| MMSE | 25 (4) | 28 (2) | < 0.001 |
| MoCA | 17 (6) | 24 (3) | < 0.001 |
| **Medications** |  |  |  |
| Anticoagulation | 2 (5.1) | 3 (4.7) | 0.920 |
| Antiplatelet therapy |  |  | 0.810 |
| No antiplatelet therapy | 2 (5.1) | 3 (4.7) |  |
| Single antiplatelet therapy | 17 (43.6) | 24 (37.5) |  |
| Dual antiplatelet therapy | 20 (51.3) | 37 (57.8) |  |
| Statins | 38 (97.4) | 59 (92.2) | 0.270 |
| Antidiabetic agent | 14 (35.9) | 22 (34.4) | 0.875 |
| Antihypertension | 25 (64.1) | 34 (53.1) | 0.275 |
| Dl-3-n-Butylphthalide (NBP) | 22 (56.4) | 42 (65.6) | 0.350 |

Continuous variables are expressed as median (IQR). Categorical variables are expressed as frequencies (percentages).

CI-L, late cognitive impairment after stroke; nCI-L, non-late cognitive impairment after stroke; ESR, erythrocyte sedimentation rate; CRP, C-reactive protein; IL-6, interleukin-6. Dual antiplatelet therapy was changed to single antiplatelet therapy after 21 days according to guidelines.
